# Plant Defense Elicitation by the Hydrophobin Cerato-Ulmin and Correlation with Its Structural Features

**DOI:** 10.3390/ijms24032251

**Published:** 2023-01-23

**Authors:** Mariana Gallo, Simone Luti, Fabio Baroni, Ivan Baccelli, Eduardo Maffud Cilli, Costanza Cicchi, Manuela Leri, Alberto Spisni, Thelma A. Pertinhez, Luigia Pazzagli

**Affiliations:** 1 Department of Medicine and Surgery, University of Parma, 43125 Parma, Italy; 2 Department of Biomedical Experimental and Clinical Sciences, University of Florence, 50121 Firenze, Italy; 3 Institute for Sustainable Plant Protection, National Research Council of Italy, Sesto Fiorentino, 50019 Florence, Italy; 4 Department of Biochemistry and Organic Chemistry, Institute of Chemistry, São Paulo State University (UNESP), Araraquara 14800-901, Brazil

**Keywords:** fungal PAMP, hydrophobins, plant defense, self-assembly

## Abstract

Cerato-ulmin (CU) is a 75-amino-acid-long protein that belongs to the hydrophobin family. It self-assembles at hydrophobic–hydrophilic interfaces, forming films that reverse the wettability properties of the bound surface: a capability that may confer selective advantages to the fungus in colonizing and infecting elm trees. Here, we show for the first time that CU can elicit a defense reaction (induction of phytoalexin synthesis and ROS production) in non-host plants (*Arabidopsis*) and exerts its eliciting capacity more efficiently when in its soluble monomeric form. We identified two hydrophobic clusters on the protein’s loops endowed with dynamical and physical properties compatible with the possibility of reversibly interconverting between a disordered conformation and a β-strand-rich conformation when interacting with hydrophilic or hydrophobic surfaces. We propose that the plasticity of those loops may be part of the molecular mechanism that governs the protein defense elicitation capability.

## 1. Introduction

Hydrophobins are low-molecular-weight amphiphilic proteins secreted as monomers by filamentous fungi. They are characterized by a moderate to a high level of hydrophobicity and by the presence of a distinctive intramolecular four-disulfide-bridge pattern. Their functionality relies on the ability to self-assemble into amphipathic layers at the interface between hydrophobic–hydrophilic surfaces [1,2,3]. 

Hydrophobins confer water-repellent properties to the fungal cell wall and play a role in some fungal functions, e.g., (i) the formation of fungal aerial structures, (ii) the attachment of hyphae to the hydrophobic surfaces of their hosts and in some cases, (iii) the capacity to become pathogenic [3,4].

The presence of the characteristic intramolecular four-disulfide-bridge pattern [1] makes these proteins globular, compact, and rather rigid. Despite the very low sequence similarity, all hydrophobins share a similar fold with a β-barrel or a half-β-barrel core and an additional α-helix stretch or a β-strand, inserted into loop two, that connects the first and second β-strands of the β-barrel [5]. The disulfide network forces some hydrophobic residues to be exposed on the protein surface, generating hydrophobic patches [6] that justify the tendency, when in contact with hydrophilic media or surfaces, to self-assemble.

As for the role of hydrophobins in fungus-plant interactions, there are controversial hypotheses. Dubey et al. [7] reported that deletion mutants for the class II hydrophobin *Hyd3* from *Clonostachys rosea* showed reduced colonization of *Arabidopsis thaliana* roots, while double *Hyd1* and *Hyd3* mutants displayed enhanced root colonization ability in comparison to the wild type. A clear role in plant root colonization emerged when the class II hydrophobin *TvHydii1* of *Trichoderma virens* was tested on *Solanum lycopersicum* plants: knock-out or overexpressing strains showed reduced and enhanced root colonization capacity, respectively [8]. In *Fusarium graminearum*, instead, single deletion strains for *FgHyd2* and *FgHyd3*, and the triple mutant *FgHyd2-3-4*, showed reduced ability to infect wheat spikelets only when using spray inoculation, suggesting that the fungus had impaired virulence because of the inability to overcome the water-air interface and attach to the hydrophobic surface of the spike tissue [9]. Recently, Cai et al. indicated intracellular hydrophobins as essential for fungal development and fitness [4].

While it is conceivable that hydrophobins can act as facilitators in symbiotic relationships between fungi and plants, the limited amount of data dealing with the ability of plants to sense the presence of hydrophobins is surprising. As it happened for the members of the cerato-platanin family soon after their discovery [10], the reason for that lack of information could be the initial classification of hydrophobins as mere toxins [11]. The ability of hydrophobins to act as plant defense elicitors is a rather unexplored field, yet.

Cerato-ulmin (CU) is a 7.6 KDa (75 residues) hydrophobin, secreted by the Ascomycete *Ophiostoma novo-ulmi*, *O. ulmi*, and *O. himal-ulmi*: fungi responsible for the Dutch elm disease (DED) [12,13,14]. The CU sequence presents the hydrophobin-conserved four-disulfide-bridge pattern and a high number of hydrophobic residues: GRAVY (Grand Average of hYdropathicity) index = 0.159 (negative GRAVY values indicate hydrophilic proteins and positive values indicate hydrophobic proteins) [15]. In addition, CU shows the typical hydropathic profile and solubility of class II hydrophobins such as HFBI and HFBII from *Trichoderma reesei*. Nonetheless, the protein self-assembles into unstable films at the interface between hydrophilic–hydrophobic surfaces: a feature that reminds of class I hydrophobins [16,17]. Overall, CU combines structural features that are intermediate between hydrophobins class I and II. Sequence similarity and hydrophobicity suggest CU is a class II hydrophobin; nonetheless, some features of CU aggregates are not consistent with that class of proteins. Class I hydrophobins give rise to highly insoluble polymers in the form of distinct rodlets, whereas class II hydrophobins produce polymers lacking rodlet organization and soluble in organic solvents [2,6,16]. A suspension of CU in water self-aggregate, under mild stirring at room temperature, produces rodlet-like structures soluble in some organic solvents [16,18].

From a biological standpoint, it is known that CU is involved in several functions vital for the fungus: it makes the hyphal surface of *Ophiostoma* species more hydrophobic, it protects the fungus from dehydration, and it may improve adhesion of the pathogen to insect vectors, thereby favoring fungal dissemination and disease spread [19]. Other studies, however, provided data against a univocal ascription of a direct role of CU in fungal pathogenicity [20]. In conclusion, the factual CU biological function has not been disclosed yet.

Here we show that CU, either in the monomeric or in the associated form, can elicit phytoalexin synthesis and ROS production, with the monomeric form inducing a faster response. Furthermore, we have identified two loops in the structure of the CU characterized by hydrophobic clusters that are expected to favor proteins association and, with dynamical features that we believe are responsible for their conformational transition from a disordered to a β-strand-like secondary structure: an initial step essential for the monomeric soluble protein to assemble in planar layers.

## 2. Results

### 2.1. Expression and Purification of rCU and U-^15^N-Labeled rCU

**Figure 1:**
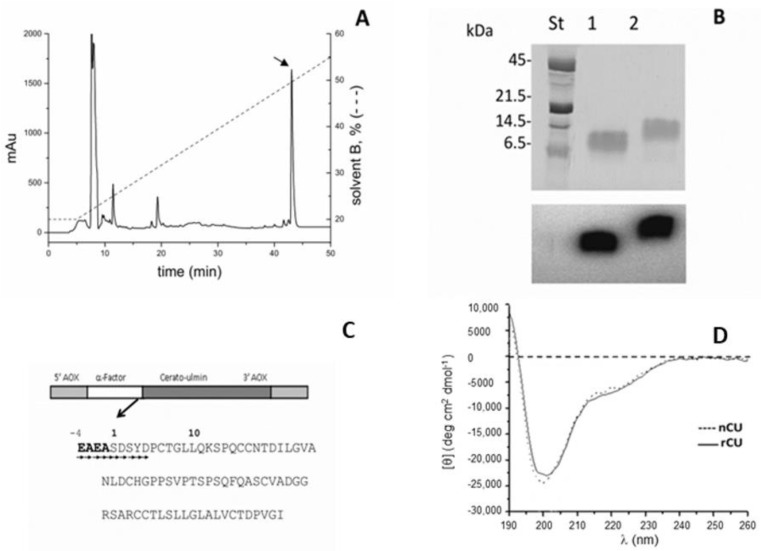
Production and characterization of recombinant CU (rCU). (**A**) HPLC purification of 500 µL culture filtrate: solvent A, 0.1% TFA in water; solvent B, 0.1% TFA in acetonitrile. The dashed line represents the elution gradient. The arrow indicates pure rCU. (**B**) SDS-PAGE (upper panel) and Western blot analysis (lower panel) of native CU (line 1) and rCU (line 2). Western blot analysis was performed with anti-CU rabbit polyclonal antiserum and detected with horseradish-conjugated secondary antibodies. (**C**) pPIC9 plasmid transformed with *cu*-gene (upper panel) and sequence of mature rCU (lower panel). Arrows indicate residues sequenced by Edman degradation to confirm N-terminus. (**D**) Circular Dichroism spectroscopy of native and recombinant CU. Far-UV CD spectra of native CU (dotted line) and rCP (solid line). Sample concentration was 6.25 µM in 30% ACN/70% H_2_O at 25 °C.

rCU was expressed in *Pichia pastoris,* as previously described [19], using the pPIC9 vector that enables secretion of the protein in the medium, thus facilitating the purification procedure: pure rCU was obtained by a single-step RP-HPLC (Figure 1A). The purity of the protein was checked by 15% SDS-PAGE (Figure 1B, upper panel) followed by Western blot analysis that confirmed rCU to be pure and with a molecular weight only slightly greater than that of the native one (Figure 1B, lower panel). Starting from 1 L of the culture’s filtrate, we obtained 9.5 mg of pure rCU. The molecular mass of purified rCU, determined by mass spectrometry, turned out to be 8019.75 Da, in accordance with the protein primary sequence (7619.58 Da) increased by the N-terminal tag EAEA (418.4 Da), originating from the restriction sites used in the expression plasmid pPIC9 (Figure 1C). The N-terminus EAEASDSYD sequence was confirmed by Edman degradation. The additional EAEA sequence, however, does not affect the secondary structure (Figure 1D).

The U-^15^N-labeled rCU was obtained following the same protocol: 4.6 mg of U-^15^N-labeled rCU was obtained from 1 L of culture filtrate. The purity of the protein was checked by 15% SDS-PAGE and confirmed by mass spectrometry analysis. The percentage of labeling was about 99.8%, as shown by mass spectrometry: m/z = 8110.75.

### 2.2. Biological Activity

As pointed out in the Introduction, since its discovery, CU has been considered a toxin [19,20]. Additionally, it has been recognized that hydrophobins can exert their biological functions both in their monomeric and associated states [21].

We tested the elicitor activity of CU as its ability to induce the production of phytoalexins, secondary metabolites typically produced by plants upon interaction with fungal pathogens and elicitors [22], using *Arabidopsis thaliana* as the plant host (see Materials and Methods).

#### 2.2.1. Native CU and Recombinant CU have Similar Elicitor Activity

The amount of phytoalexins produced in the lower surface of the leaves, when in contact either with native (nCU) or rCU, is comparable: 7.5 ± 0.9 and 8.1 ± 1.3 nmol/mL, for nCU and rCU, respectively.

Having verified that nCU and rCU exhibit similar biological activity, we carried out the experiments using rCU, which can be conveniently produced in large amounts.

#### 2.2.2. Comparison of the Biological Activity of rCU in Monomeric and Associated Forms

Vigorous shaking of the CU water solution leads to an increase in turbidity, indicating protein association, as previously known, and reported in the Material and Methods section. To detect the protein biological activity in that state, right after the solution became milky, drops of that suspension were deposited on the lower surface of *Arabidopsis* leaves. In parallel, drops of the protein in its monomeric state, in a 30% ACN/70% H_2_O solution, were applied to a second set of leaves. Control experiments were performed using 30% ACN/70% H_2_O to detect any effect due to the medium.

**Figure 2:**
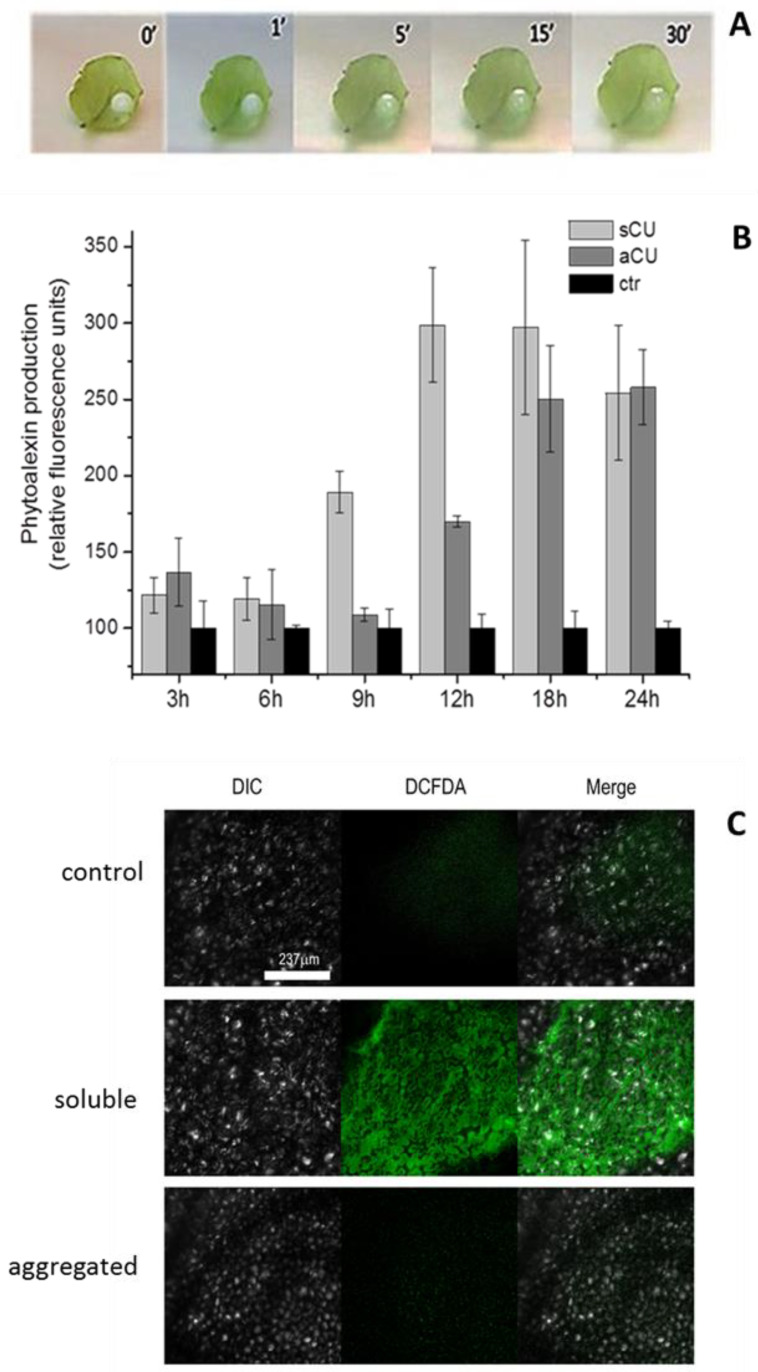
Effect of monomeric and self-assembled rCU on *Arabidopsis t.* leaves. (**A**) Optical photographs at various time intervals of droplets of aggregated rCU in water applied on the lower surface of *Arabidopsis* leaves. (**B**) Time course of phytoalexins production. Leaves were treated on the lower surface with 10 μL droplets of 40 µM rCU in 30% ACN/70% H_2_O (soluble/monomeric CU, sCU, light grey); 10 μL droplets of 40 µM in water aggregated rCU (aCU, dark grey) and with 10 μL droplets of 30% ACN/70% H_2_O (control, black). Drops were collected after 3, 6, 9, 12, or 24 h of treatment; phytoalexins release was measured by fluorescence analysis (λ_ex_ = 320 nm, λ_em_ = 386 nm). The fluorescence value was normalized to the number of droplets analyzed and expressed as relative fluorescence units. Error bars indicate standard deviations of three biological replicates performed in duplicate. Statistical analysis was performed by unpaired t-test (treated vs. control). (**C**) Synthesis of H_2_O_2_. H_2_O_2_ is visualized in situ by the fluorescent probe DCFH2-DA. Fluorescence was detected at a *λ*_ex_ of 488 nm under a confocal Leica TCS SP5 scanning microscope, and photographs were obtained of representative regions of three different leaves treated with rCU for 9 h. DIC: Differential interference contrast of leaves. DCFDA: fluorescence recovered from leaves at 488 nm. Merge: merging of the DIC and DCFA layers.

Interestingly, we noted that 30 min after deposition on the leaf surface, droplets of the associated proteins lost their milky appearance and turned transparent (Figure 2A).

The biological activity of CU in the two states was evaluated, as described in Material and Methods, by measuring the production of phytoalexins and H_2_O_2_ via fluorescence tests. Measurements were carried out at 3 h intervals, starting after the first 3 h from proteins deposition up to 24 h. In all tests, we could not detect any fluorescence for incubation times shorter than 3 h due to the time necessary for phytoalexins synthesis. The fluorescence recovered from the droplets, Figure 2B, indicates that the increase in the immune response elicited by the soluble rCU in the initial 6–18 h is higher than the one observed for the self-assembled rCU. After 18 h, the difference in the intensity of the response decreases, and at 24 h, the intensity of the defense response for the soluble and aggregated rCU is comparable.

One of the key events characterizing the signaling cascade in plant defense is the production of Reactive Oxygen Species (ROS). Therefore, the ability of rCU to induce ROS production was assayed as the synthesis of H_2_O_2_ induced by both the soluble and aggregated protein after 9 h of droplets deposition, visualized in situ by the fluorescent probe DCFH2-DA. The images (Figure 2C) clearly show that H_2_O_2_ produced after 9 h is more abundant in leaves treated with monomeric rCU than in those treated with the aggregated one, thus confirming the previous results.

In summary, the results indicate that though the monomeric and aggregated rCU, in the 18–24 h of incubation, elicit an immune response of comparable intensity, the monomeric form induces a faster response. We hypothesize that after the interaction of the aggregated proteins with the leaves’ hydrophobic surface, there is a time interval necessary for the release of the monomeric soluble form: the one able to penetrate through the foliar epidermis.

### 2.3. CU 3D Model

**Figure 3:**
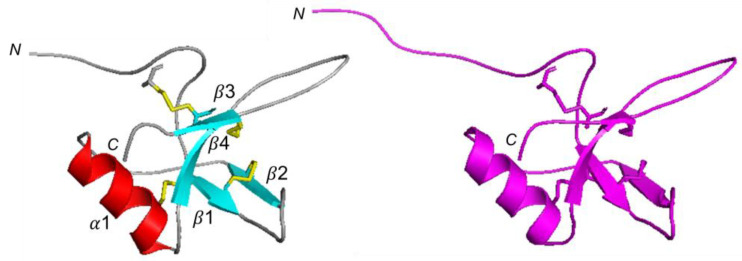
CU 3D NC2-based model. (**A**) CU 3D model built using NC2 as a template and (**B**) 3D structure of NC2 (PDB 4AOG). The calculated backbone atoms RMSD between the two structures is 0.60 Å. Boxed numbers indicate the disulfide bridges.

Despite the low sequence similarity, hydrophobins share a similar fold; thus, we constructed a CU 3D model using the server SwissModel [23] and, as a template, the NMR structure of the class II hydrophobin NC2 (PDB 4AOG, [21]; NC2/CU sequence identity 44.1%), that, among the various hydrophobins structures in the PDB, provided the best quality model for our purpose. The calculated structure (Figure 3) shows four antiparallel β-strands (β1, residues 17–19; β2, residues 28–31; β3, residues 54–58, β4 residues 69–71) and an α-helix (α1 residues 40–49). The four disulfide bridges (C7–C57; C17–C47; C18–C30; C58–69) stabilize the fold. The stereochemical quality of the model was further verified by PROCHECK [24].

To validate the calculated structure, we carried out a suite of NMR experiments using ^15^N-labeled rCU: (i) Diffusion experiments provided a hydrodynamic radius (R_h_) = 14.2 ± 0.4 Å, which agrees with the value calculated from the chosen PDB model structure, R_h_ = 16 Å, using the Hydropro software [25] and indicates that the protein is in a monomeric state. (ii) the good quality of the ^1^H–^15^N HSQC spectrum, characterized by an excellent chemical shift dispersion of the signals and rather homogeneous linewidths, further confirmed the presence of a well-folded monomeric protein without any evidence of aggregation (Figure S1, Supplementary Material). All the 71 expected backbone amide resonances of non-proline residues were detected and, by combining 2D and 3D NMR experiments, were assigned. (iii) Analysis of backbone chemical shifts using TALOS+ [26] predicted secondary structure elements in agreement with the model. Similarly, the long- and medium-range NOEs turned out to be consistent with the CU calculated model. (iv) NMR H/D exchange and CLEANEX-PM plots confirmed that amides located in secondary structure elements were protected from the solvent exchange (Figure S2, Supplementary Material). (v) A total of 59 experimental residual dipolar couplings (^1^D_HN_, Table S1, Supplementary Material) were fitted to the model. From the analysis, we excluded the N-terminal residues up to D5 because of their high flexibility (see below) and L11, V25, C57, and V73 due to the signals’ overlap. The correlation factor, expressed as the Q_factor_, turned out to be Q_factor_ = 0.56 (Figure S3, Supplementary Material). The Q_factor_ approximates to zero in case of perfect agreement between experimental and calculated data. In the medium-resolution crystal structure, generally, it scores around 0.3, and in NMR-resolved structures below 0.2. Values around 0.7 have been reported in the case of very flexible regions [27]. Because we are dealing with a theoretical model, where 72% of the residues are in disordered regions, the obtained value indicates the model can be considered representative of the real structure.

The validated CU 3D model and the NMR-derived structure of NC2 present a similar core characterized by an open β-barrel and two loops extending from the protein. The main difference is in the N-terminal region that, in CU, is shorter, Figure 3.

### 2.4. CU Aggregation/Biofilm Formation

**Figure 4:**
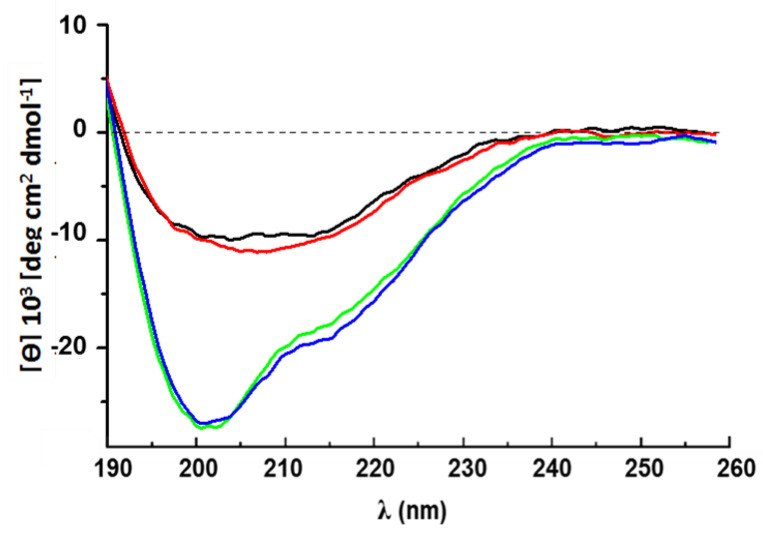
Far-UV CD spectra of rCU. CD spectra of 10 µM rCU in different conditions: (i) 30% ACN/70% water (green line); (ii) 30% ACN/70% 50 mM Na-phosphate buffer (blue line); (iii) rCU suspended in water and then dried on a quartz cuvette window (red line); rCU adsorbed on colloidal Teflon (black line).

The CD spectra of rCU in solution, whether in 30% ACN/70% H_2_O or in 30% ACN/70% 50 mM Na-phosphate buffer pH 5.8 (Figure 4), are dominated by contributions from disordered regions (minimum at ∼200 nm). However, the negative shoulder that spans the region from 218 to 225 nm is indicative of the presence of secondary structure elements, β-strands, and short helical regions, in agreement with the calculated model. Instead, when the protein is dried onto the wall of a quartz cuvette or absorbed on colloidal Teflon, the CD spectrum presents a single wide negative band centered at ∼210 nm and a positive one, presumably, at ∼190 nm, indicating an increase in β structure. The possible formation of CU β-amyloid-like aggregates is supported by a positive reaction to Thioflavin T [16].

Because RFAmyloid, a web server predicting amyloid proteins [28], classifies CU as an amyloid protein with 80% probability, using AGGRESCAN [29], we searched for regions with a propensity for β-aggregation, and we identified the N-terminal loop (residues T8-Q12), and the loops between strands β1–β2 and β3–β4 including part of the β3- and β4-strands (Figure S4, Supplementary Materials).

### 2.5. rCU residues Involved in Slow Conformational Motions

A complete set of backbone ^15^N relaxation data (R_1_, R_2_, and hetNOE) was acquired to investigate the internal dynamics of rCU on both ps–ns and µs–ms time scales.

Excluding residues with overlapping or very weak correlation peaks and prolines, we measured relaxation data for 64 out of 71 residues (Figure S5, Supplementary Material). The first eight residues in the N-terminal segment (comprising the additional non-native EAEA sequence, residues −4 to −1), which exhibit hetNOEs < 0.5, undergo high conformational flexibility. The protein core, instead, is rather rigid as the disulfide network could anticipate; backbone average R_1_ = 1.61 ± 0.23 s^−1^, R_2_ = 11.0 ± 4.7 s^−1^, and hetNOE = 0.77 ± 0.05. Nonetheless, some core residues show reduced hetNOEs (< 0.7), indicating flexibility in the ns–ps time scale: Q12 in the N-terminal loop; the stretch T20-N27, in the β1β2-loop; residues D29-C30, in the β2-strand; V36, in the β2α1-loop; L63 and A66-V67, in the β3β4-loop; and G74-I75, in the C-terminus. Consistently, residues Q12 (N-terminal loop), D21 (β1β2-loop), and the stretch G74-I75 experience R_2_ values lower than the average. Residues with R_2_ higher than the average, instead, are candidates to be involved in slow conformational motions: K13, near the N-terminus of β1-strand; I22 and A26-N27, in the β1β2-loop; L28 and C30, in the β2-strand; and V68, in the β3β4-loop.

**Figure 5:**
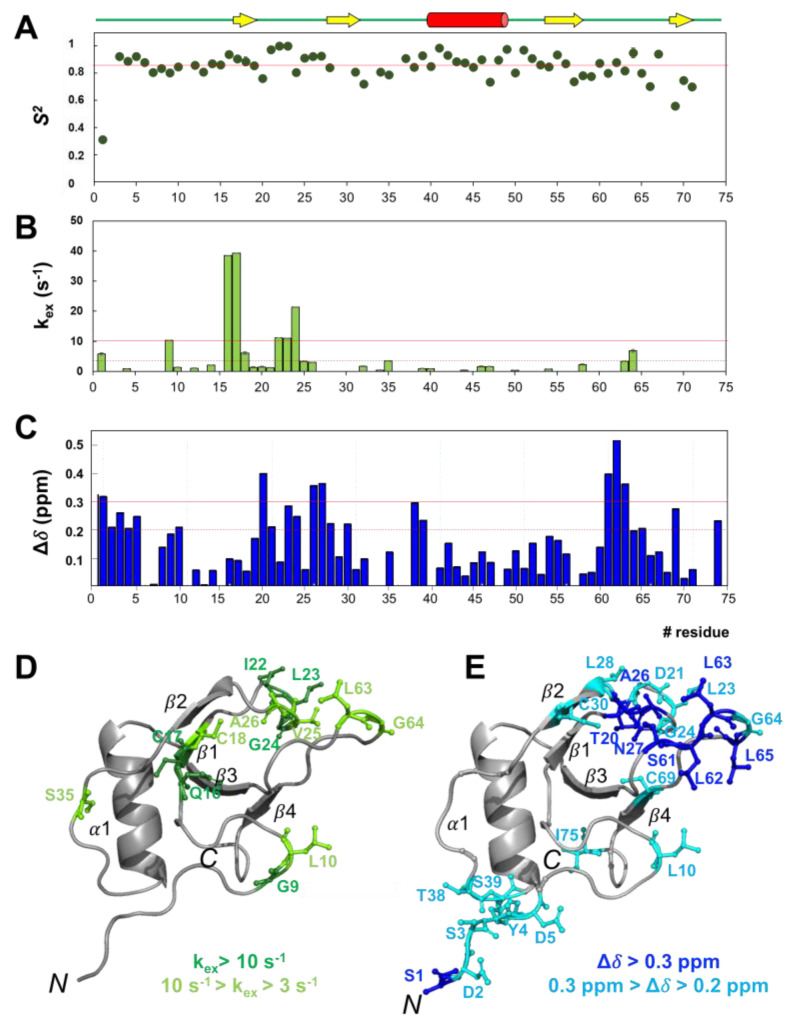
CU residues involved in slow conformational motions and residues with conformation sensitive to the polarity of the medium. (**A**) Lipari–Szabo model-free analysis. The order parameter *S*^2^ is plotted as a function of the residue number. The red line indicates the average *S*^2^ value (top panel). Conformational exchange contributions, k_ex_ values of 3 and 10 s^−1^ are displayed as full and broken red lines, respectively (lower panel). (**B**) CSP analysis upon ACN: phosphate buffer titration. ∆δ values of 0.2 and 0.3 ppm are displayed as full and broken red lines, respectively. (**C**) Residues with significant k_ex_ are mapped onto the CU model. (**D**) Results from the CSP are mapped onto the CU model. (**E**) Results from the CSP are mapped onto the CU model.

The NMR relaxation data were analyzed by the model-free formalism [30] using the TENSOR2 program. The anisotropic model of diffusion was used based on the axial ratio, a/b = 1.69, obtained from the PDB model of the CU structure using the HullRad server [31]. The calculated overall rCU correlation time, τ_c_ = 7.2 ns, agrees with the presence of a monomeric and rather compact protein. The order parameter S^2^, ranging from 0 for completely unrestricted motion to 1 for a completely rigid residue with respect to the molecular reference frame, is sensitive to flexibility on the ps–ns time scale. The rCU core has a relatively flat profile with an *S*^2^ average of 0.85 (Figure 5A top panel), confirming the overall rigidity of the protein. Residues with a fast dynamic (S^2^ < 0.75) are G32, C47, C57, and A66, and the ones in N- and C-terminus. For some residues, it has been necessary to introduce the parameter k_ex_, a term reflecting the contribution of slow conformational motions on the μs–ms time scale: residues G9 N-terminus; Q16-C18, β1-strand, I22-G24, β1β2-loop, S35, β2α1-loop, and L63−G64, β3β4-loop (Figure 5A lower panel). Those residues are mapped onto the protein molecular 3D model in Figure 5C. Quite a few of those slow-moving residues are coincident with the ones predicted to have a high propensity for β-aggregation by AGGRESCAN (Figure S4, Supplementary Materials).

### 2.6. Identification of Key Residues Involved in Self-Assembly

Hydrophobic interaction is a key driving force for hydrophobins’ self-assembly [21]. The ConSurf server [32], used to map the evolutionarily conserved residues onto the CU 3D model, searched over 150 hydrophobins and highlighted the cysteine network, hallmark of that protein family, L11 and some hydrophobic residues located in the β1β2- and β3β4-loops (Figure S6A,B, Supplementary Materials). Consistent with previous studies [6], Figure S6C highlights the existence of the hydrophobic region, highly conserved amongst all members of the family, that we hypothesize may be involved not only in protein association but also in protein surface interaction.

To support that hypothesis, we investigated the protein ^1^H chemical shift perturbation (CSP∆δ) upon ACN:phosphate buffer titration; Figure S7, Supplementary Materials, reports the CSP for some selected residues. Knowing that in pure ACN and in 30% ACN:70% 50 mM Na-phosphate pH 5.8 buffer rCU is soluble and monomeric while in water, the protein aggregates, the experiment will highlight the residues affected by the change in the medium polarity and that are more likely to be involved in the self-assembly process. The complete CSP analysis (Figure 5B,D) shows that, amongst the residues with ∆δ > 0.2 ppm, there are (a) residues belonging to loops β1–β2 and β3–β4 that are part of a hydrophobic patch suggested as responsible for protein aggregation [6], as well as residues that are amongst the conserved ones in the hydrophobin family (Figure S6A,B, Supplementary Material): T20-D21, L23-G24, and A26 (loop-β1 β2); S61 and L65 (loop-β3 β4). Additionally, there are (b) several residues corresponding to the ones identified by AGGRESCAN (Figure S4, Supplementary Materials): L10 (N-terminus); D21, L23-G24, and A26 (loop-β1 β2); S61-L65 (loop-β3 β4); and C69 (β4-strand). Lastly, there is (c) a number of residues involved in slow conformational dynamics (Figure 5A (lower panel), C).

## 3. Discussion

In 1974, Takai identified a new metabolite from *Ophiostoma ulmi* (formerly *Ceratocystis ulmi*) that he named cerato-ulmin [14]. Since then, CU has been considered a wilt toxin produced by DED pathogens because of its ability to cause, in elms, symptoms comparable to those occurring during DED: drooping of leaves followed by irreversible wilting, chlorosis, and eventually necrosis [14]. As reviewed by Temple and Horgen [33], only around the end of the nineties it became evident that CU was required for the pathogenicity of DED pathogens. Nevertheless, the reasons behind the capacity of CU to act as a “wilting inducer” on leaves have remained elusive.

In the present study, we investigated the CU eliciting activity on the model, non-host plant *A. thaliana* by applying rCU on *Arabidopsis* leaves, and we found that the protein induces the synthesis of antimicrobial molecules such as phytoalexins and causes the production of ROS (namely H_2_O_2_) (Figure 2): a trademark of plant immune responses induced by MAMPs (microbe-associated molecular patterns) [22,34,35]. Because those defense-related responses are exhibited also by other small fungal Cys-rich secreted proteins named cerato-platanins [10,36,37], it is conceivable to hypothesize that CU is perceived by *Arabidopsis* leaves as a MAMP, thus opening a new scenario for the role of CU in DED pathogens and likely explaining the wilting activity of the pure protein on leaves. Recalling that purified native CU interacts, in vitro, with cultured elm cells and causes dose-dependent cell death [38], we assume that CU is a hydrophobin possessing MAMP-like activity. This hypothesis is supported by a study on the hydrophobin Hytlo1 from *T. longibrachiatum*, suggesting it plays multiple functions in plant–fungus interactions, displaying direct antifungal, eliciting, and plant-growth promotion activities [39].

Having verified that (i) CU self-assembles in ordered aggregates that react with Thioflavin T and revert, with time, to the monomeric form, and (ii) both the monomeric and aggregated forms induce defense responses, with the monomeric form triggering a more rapid immune reaction, we searched for structural features that may provide a model for the molecular mechanisms associated to the observed in vitro biological activity.

According to the hypothesis that the main driving force in the oligomerization of hydrophobins is hydrophobic interactions of specific regions exposed on the protein surface [6], we searched for residues that might be involved in the assembling process. Combining AGGRECAN analysis, Figure S4, Supplementary Materials, with an NMR CSP study, Figure 5B,D, we identified two hydrophobic clusters on loops β1–β2 (D21, L23-G24, and A26) and β3–β4 (S61-L65). The hydrophobic surface patch also includes L10 in the N-terminus (Figure S6C, Supplementary Materials). H/D exchange and CLEANEX-PM NMR experiments (Figure S2, Supplementary Materials) confirmed that those residues are exposed to the solvent and are prevented from burying their sidechains inside the protein core by the sulfur bridges network. The NMR relaxation data (Figure 5) revealed that despite CU being a rigid molecule, the regions spanning the hydrophobic patch present a higher degree of flexibility, especially in the slow conformational μs–ms time scale. It is, therefore, reasonable to hypothesize that the conformational dynamics of those solvent-exposed hydrophobic regions may play a key role in protein–protein and protein–surface interactions.

### A Molecular Model for CU Biological Activity in Vitro

As shown in Figure S6C, the protein exhibits hydrophobic and hydrophilic regions of different extents, the hydrophobic ones being more extended: 70% of the solvent-accessible surface area of the protein is hydrophobic according to GetArea [40]. Those surface features allow the proteins to exist, depending on the medium polarity, either in a monomeric or in a self-assembled state. When drops of a 30% ACN/70% H_2_O solution containing the protein in its monomeric state are laid on the leaves’ surface, the protein can diffuse from the drop to the water layer that covers the leaves’ hydrophobic surface. Soon after, however, the hydrophobic surface of the leaves will exert a stronger attraction producing the exit of the protein from the solution and its binding to the leaves’ surface. The process is favored by the flexibility of the hydrophobic regions that, as highlighted by the CD spectra in Figure 4, acquire an organized secondary structure, primarily β-sheet, thus being able to bind tighter to the surface and subsequently penetrate the host.

Although the importance of hydrophobin aggregation is recognized as relevant for their biological activity [1,4,11], here we show that, in eliciting plant defenses, CU, when in its self-assembled form, is less competent than in the soluble form.

**Figure 6:**
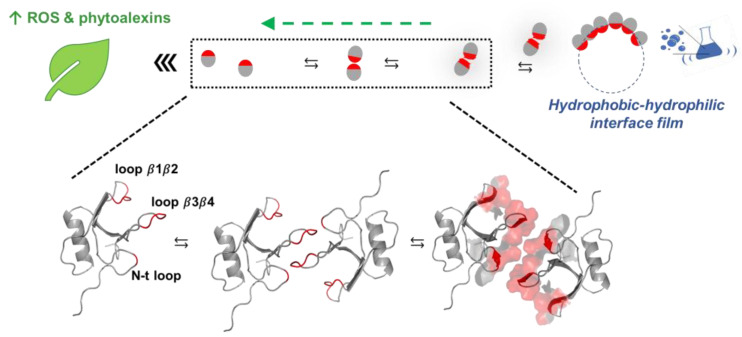
Model for CU biological activity in vitro.

Though the assembling process of the protein is not completely understood, what is known is the ability of CU to stabilize air bubbles and oil droplets through the formation of a protein film on the external surface [41]. It is, therefore, reasonable to envisage that the protein interacts with the air bubbles produced by the vigorous shaking and assembles on their hydrophobic surface forming a layer that exposes the hydrophilic protein regions. Having verified that the transition from the soluble to the associated state is reversible (Figure 2), we hypothesize that the associated proteins present on the drop of the milky solution, once laid on the leaves’ surface, slowly revert to their monomeric state and then follow the process described before (Figure 6).

This model fits with the observed reduced rate of production of phytoalexins and ROS induced by the aggregated form of CU, in the first 18 h, in comparison with the monomeric one (Figure 2B).

In conclusion, here we show that, when dealing with proteins, the combination of biological and structural data is a key strategy to deepen the understanding of the molecular mechanisms that underly their functionality.

We believe that the in vitro model we have presented will stimulate new ideas to better understand the role, in vivo, of hydrophobins in the pathogenic fungi-plant interaction process.

## 4. Materials and Methods

### 4.1. Native Cerato-Ulmin (nCU) and Recombinant Cerato-Ulimin (rCU) Production

The nCU protein has been isolated from liquid culture of O. novo-ulmi 182, where it exists in a soluble form, according to the method described in Sbrana et al. [16]. The protein purity and molecular weight were checked by SDS-PAGE and MALDI-TOF mass spectrometry.

The rCU protein was obtained by cloning and expressing the *cu* gene in *Pichia pastoris* [42]. Standard molecular biology protocols were used for cloning and expression. The pGEMT Easy plasmid from *E. coli* DH5α cells containing the cu gene was used to isolate the cDNA fragment. The *E. coli* DH5 α (Invitrogen) was used for routine plasmid amplification; the pGEM-T Easy vector was purchased from Promega (Madison, WI, USA). Total RNA was extracted from a liquid culture of *Ophiostoma novo-ulmi* MH75 using TRI REAGENT (Sigma) and purified by LiCl precipitation. Since the encoding cu gene (Accession n° AJ519672) contains two introns of 76 and 75 bp, respectively, total RNA was used to perform RT-PCR and PCR experiments. Primers (CUcodFor and CUcodRev) were designed based on the secreted protein sequence, excluding the signal peptide (CUcodFor: AGCGACTCCTACGACCCTTGCAC; CUcodRev: TTAAATGCCGACGGGGTCGGTGC). *Cu* gene was amplified with the primers XhoICUFor (CTCGAGAAAAGAGAGGCTGAAGCT) and EcoCURev (GAATTCTTAAATGCCGACGGGGTCG). PCR amplification was performed with a Gene Amp PCR System 2400 from PerkinElmer (Wellesley, MA, USA). A 1 Kb plus DNA Ladder (Invitrogen) was used for 1% agarose gel electrophoresis. The PCR product was subcloned into pGEM-T easy vector. The pGEM-cu was purified by ethanol precipitation and electroporated into *E. coli* DH5α using a Gene Pulser II (Bio-Rad). *E. coli* cells were grown in SOC (Super Optimal broth with Catabolite repression; Sigma-Aldrich, Italy) medium and then selected on LB (Luria broth) dishes. *E. coli* DH5α cells were screened for the presence of the cu gene by the blue/white test. Positive colonies were analyzed by colony PCR using the above primers, and sequences were confirmed by the Bio Molecular Research Lab. c/o CRIBI, University of Padua, Italy. The pGEM-cu was extracted from an overnight liquid culture of one positive clone. Approximately 60 µg of plasmid was linearized with the Bgl/II restriction enzyme, the digestion products were purified from salt and enzymes, and the construct was digested with EcoRI and XhoI and cloned into the same enzyme digested vector pPIC9 using T4 DNA ligase. The pPIC9 vector carrying the secretion signal of the α-mating factor sequence from *S. cerevisiae* was chosen to enable the secretion of the expressed protein. Restriction enzymes, T4-DNA ligase, and Taq DNA polymerase were purchased from Invitrogen (San Diego, CA, USA). The host P. pastoris strain GS115 (his4) from the *Pichia* expression kit was transformed with cu-pPIC9 vector and analyzed according to the manufacturer’s instructions.

Approximately 60 µg of plasmid were linearized with the Bgl/II restriction enzyme; the digestion products were purified from salt and enzymes by extraction with phenol/chloroform/isoamyl alcohol (25/24/1) and precipitated with ethanol. The obtained fragments were suspended in water and used to transform *P. pastoris* GS115 His-cells, made competent according to the manufacturer’s instructions. Transformants were screened for methanol utilization by patching about 80 colonies on minimal dextrose (MD) and minimal methanol (MM) dishes, incubated for 3 days at 30 °C, and scored as MutS (methanol utilizing slow) or Mut+ (methanol utilizing plus). Integration of cu into the *Pichia* genome was confirmed by colony PCR. Mut+ integrants produce two bands, while Mut S integrants show only one band corresponding to cu gene cloned into pPIC9.

Subsequently, four clones of the His + Mut + transformed strains were selected: each clone was patched on an MD dish and incubated at 30 °C for 3 days. A single colony of each clone was used to inoculate 100 mL of MGY (minimal glycerol medium) in a one-liter flask and grown at 30 °C in a shaking incubator until the cultures reached an OD of 6.0 at 600 nm. rCU expression was induced by adding methanol daily for 4 days at 28 °C.

Aliquots (500 µL) of the expression cultures were centrifuged at 8000 g for 5 min at different times. Both pellets and supernatants were subjected to 15% SDS-PAGE to choose the colony that best expresses rCU.

Large-scale production was started by patching the colony on MD and incubating it at 30 °C for 3 days. A single colony was then inoculated in 1.0 L of MGY until the OD at 600 nm was 6.0. After centrifugation at 3000 g for 15 min, the pellet was re-suspended in 100 mL of MM, placed in a 1.0 L flask, and returned to the incubator for further growth. Every 24 h, 100% methanol (15 µL) was added to maintain induction, and after six days, the medium was centrifuged (8000 g for 15 min), and the supernatant was concentrated by ultra-filtration on a YM3 Millipore membrane.

rCU was purified, in soluble form, from the liquid culture by RP-HPLC using a semi-preparative reverse phase HPLC column (Phenomenex, C4, 5 µm, 250 × 10 mm). Elution was performed with a water/acetonitrile gradient in 0.1% (*v*:*v*) trifluoroacetic acid. Peaks containing the protein were collected, freeze-dried, and re-dissolved in 30% acetonitrile (ACN) to maintain the CU in the soluble form.

Purified rCU was quantified by bicinchoninic acid assay (Pierce) and checked by mass spectrometry. Twenty picomoles of either nCU or rCU were dissolved in 50% acetonitrile containing 0.05% TFA, diluted 1:1 in a sinapinic acid matrix. The protein molecular weight was determined by MALDI–TOF mass spectrometer (Omniflex, Bruker-Franzen Analytik), the N-terminal sequence was obtained by Edman degradation, on a Procise model 491 gas-phase protein sequencer (Applied Biosystems), after reduction and carboxymethylation according to [42]. nCU and rCU were subjected to 15% SDS-PAGE electrophoresis using protein markers for reference and Coomassie blue for staining (Bio-Rad, Hercules, CA, USA). One of the obtained gels was subjected to Western blot analysis using a polyvinylidene difluoride (PVDF) membrane for the transfer. Native and recombinant CU were detected using an anti-CU antiserum raised in rabbits against purified CU from culture filtrates of *O. novo-ulmi* isolate H328 [13]. The blotted membranes were washed three times and incubated with horseradish peroxidase-conjugated secondary anti-rabbit antibodies (Cell Signaling) diluted 1:2000 in PBS containing 5% BSA and 0.1% Tween 20 for 1 h. After successive washing, the membranes were developed using an ECL kit (GE Healthcare). Chemiluminescence signals were acquired with a molecular imaging station from Kodak.

To produce ^15^N uniformly labeled rCU (U-^15^N-labeled rCU), a single Mut+ colony was used, grown in culture medium containing (^15^NH_4_)_2_SO_4_, and the protein was purified as reported above. Protein concentration was determined by Bicinchoninic acid assay, and ^15^N-labeled rCU molecular mass was determined by MALDI-TOF mass spectrometry using 20 pmol of the ^15^N-labeled protein following the protocol described for rCU characterization.

### 4.2. Induction of Aggregation of nCU and rCU

nCu and rCU exist in soluble form as long as they are in the culture medium. Once purified, they are maintained in their monomeric form in 30% ACN/70% H_2_O. Aggregation of both nCU and rCU is induced (starting from a solution of the protein in H_2_O) by continuous stirring overnight in a balancing shaker at 50 rpm/min in H_2_O at 25 °C according to Sbrana et al. [16]. Turbidity of the protein solution after stirring is considered an index of protein association [16]. Once the aggregation state is reached, the protein dispersion, characterized by a milky appearance, is immediately used for the detection of its biological activity.

### 4.3. Evaluation of the Biological/Elicitor Activity

The biological activity of nCU and rCU was determined as the ability to induce phytoalexin synthesis on *Arabidopsis* leaves, according to Baccelli et al. [43]. Briefly, 40 µM of nCU or rCU in 30% ACN/70% H_2_O were applied (as 10 µL droplets) on the lower surface of *Arabidopsis* leaves. After 24 h, droplets were recovered, and the fluorescence was measured with a PerkinElmer fluorimeter 650-10S (PerkinElmer, Wellesley, MA, USA), λex 320 nm, λem 386 nm, and slit 5. The results were expressed as nmol/mL of umbelliferon equivalents. Data obtained were analyzed by ANOVA. Homogeneous groups were identified by means of Tukey’s HDS test. Control values were obtained by treating leaves with a 30% ACN/70% H_2_O solution without the protein.

Due to the limited amount of the native protein and because we verified that nCU and rCU behave similarly (see Results section on “Biological Activity”), the biological activity of aggregated CU was determined using rCU only. As previously described, according to Baccelli et al. [42], 10 µL of a 40 µM milky solution of rCU in 50 mM Na-phosphate buffer pH 5.0 were applied on the lower surface of *Arabidopsis* leaves. Treated leaves were put into moist Petri dishes and incubated under continuous light at room temperature. Drops were collected from the lower leaf surface after 3, 6, 9, 12, 18, and 24 h of incubation, and the phytoalexin measurement was performed by fluorescence analysis (λex = 320 nm, λem = 386 nm, slit 5) using a PerkinElmer spectrofluorimeter 650-10 S (PerkinElmer, Wellesley, MA) and reported as fluorescence intensity/number of recovered droplets.

Data obtained were analyzed by ANOVA; homogeneous groups were identified by means of Tukey’s HDS test.

On *Arabidopsis* leaves, we also tested the ability of rCU to induce H_2_O_2_ synthesis. Samples were treated as reported above for the phytoalexin measurements. After 9 h of incubation, drops were removed, and H_2_O_2_ was visualized, in situ, by the specific probe 2-7-dichlorodihydrofluorescein-diacetate (DCFH_2_-DA; Sigma), which is rapidly oxidized to highly fluorescent dichlorofluorescein (DCF) in the presence of H_2_O_2_. After staining for 1 h, samples were washed twice in fresh 50 mM Na-phosphate buffer pH 5.0 to remove excess fluorophore and mounted on slides. Fluorescence was then observed at a λex of 488 nm under a confocal Leica TCS SP5 scanning microscope (Leica, Mannheim, Germany).

### 4.4. Nuclear Magnetic Resonance (NMR) Spectroscopy and Modeling

NMR experiments were recorded at 15 °C on a Varian Inova spectrometer operating at 14.09 Tesla, 600 MHz for the ^1^H resonance, and equipped with a z pulsed-field gradient unit and a triple-resonance probe.

NMR samples contained 700 μM of ^15^N-labeled rCU in 30% ACN-d_3_: 70% 50 mM Na-phosphate buffer pH 5.8 and TMS (Tetramethylsilane) as an internal reference unless otherwise indicated. NMR data were processed and analyzed using NMRPipe [44] and NMRView [45] software.

#### 4.4.1. NMR Diffusion Experiments

Pulsed-field gradient (PFG) self-diffusion measurements were performed using the PG-SLED sequence [46]. Sample: 350 µM U-^15^N-labeled rCU in 30% ACN-d_3_/70% 50 mM Na-phosphate buffer pH 5.8. Dioxane (20 µL 1% in D_2_O) was added as an internal standard (Rh, dioxane = 2.12 Å) [47]. The hydrodynamic radius (Rh) was determined by fitting the intensities of the protein and dioxane signals to an exponential function of the gradient strength using dioxane as a reference [48].

#### 4.4.2. Chemical Shift Assignment

Backbone and aliphatic side-chains signals were assigned by 2D ^1^H–^15^N HSQC, 3D-TOCSY-HSQC (50 ms and 60 ms mixing times), 3D-NOESY-HSQC (70 ms and 100 ms mixing times), 2D-TOCSY (50 ms and 60 ms mixing times), and 2D-NOESY spectra (70 ms and 100 ms mixing times).

#### 4.4.3. Model Construction, ^1^H–^15^N Residual Dipolar Coupling (D_HN_) Measurements and Model Validation

The Alignment Mode Modeling protocol of SwissModel [23] was used to generate 3D models of cerato-ulmin. The best model was obtained using NC2 (PDB 4AOG) as a template. The stereochemical quality of the structure was assessed with the program PROCHECK [24]. The model has been deposited in BMRB with code 51731, deposited data files: [‘C_ulmin.str’].

The CU, NC2-based model was consistent with the long- and medium-range NOE correlations observed in the NOESY spectra of rCU. The model was further validated through the fitting of experimental D_HN_ residual dipolar couplings (RDCs) [49]. D_HN_s were measured using IPAP ^1^H–^15^N HSQC experiments [50] acquired in the presence and absence of filamentous phage Pf1 (Asla Labs) [51] and analyzed through the BestFit module of the software PALES [52]. Quality of the fitting has been evaluated by calculating a quality factor Q according to the equation developed by Cornilescu et al. [53]:

$$Q=\frac{RMS\;(D_{HN}^{obs}-D_{HN}^{calc})\;}{RMS\;(D_{HN}^{obs})}$$

The 3D model was prepared using the visualization software Swiss PDB-Viewer.

#### 4.4.4. H/D Exchange and CLEANEX Measurements

For the H/D exchange experiments, the NMR sample was quickly frozen in liquid nitrogen, lyophilized, and then re-suspended in D_2_O. Soon after, a series of ^1^H–^15^N HSQC spectra were collected to monitor the decay of the ^1^H signals due to ^1^H/D exchange. ^1^H–^15^N HSQC spectra were collected every 20 min for 66 h. Exchangeable amide protons were also identified by 2D (CLEANEX-PM)-HSQC experiment [54] using a mixing time of 150 ms. Amide protons accessibility was measured as the ratio of the peak volumes of the CLEANEX-PM and ^1^H–^15^N HSQC spectra.

#### 4.4.5. ^15^N Relaxation Measurements and Backbone Dynamics

T1, T2, and ^15^N–{^1^H} NOE (hetNOE) experiments were performed using standard pulse schemes [55], as described [56]. Relaxation delays of 10, 110, 210, 410, 710, 910, and 1310 ms were employed for T1 measurements, and 10, 30, 70, 110, 150, 190, 230, and 250 ms for T2 measurements. The longitudinal (R1) and transversal (R2) relaxation rates (1/T1 and 1/T2, respectively) were extracted by fitting the time dependence of the peak intensities to a single exponential decay function using the rate analysis routine of NMRView [45]. Uncertainties of peak intensities were evaluated using the standard deviation of the spectral noise, measured in a region free of cross peaks.

HetNOE values for each residue were taken as the ratio of the peak volumes of the experiments recorded with and without ^1^H saturation using a saturation time of 5 s and a recycle delay of 5 s. Errors were estimated from the baseline noise in the two spectra. Typically, errors were of the order of 3% for T1 and T2 and 5% for hetNOE.

Model-free analysis of the relaxation data [30] was performed with the program TENSOR2 [57]. The anisotropic model of diffusion was used based on the 1.69 axial ratio a/b obtained for the PBD model of the CU structure using the HullRad server [31]. By excluding residues from −4 to 4 due to their higher mobility (hetNOE < 0.5), an overall correlation time (τc) of 7.2 ns was calculated from the experimental T1/T2 ratios. The model-free parameters for each residue were then obtained by fitting the complete set of nitrogen relaxation data (R2, R1, and hetNOE).

#### 4.4.6. Chemical Shift Perturbation upon Acetonitrile and Phosphate Buffer Titration

Chemical shift perturbation (CSP) due to the variation in the dielectric constant of the solvent, was followed by 2D ^1^H–^15^N HSQC spectra, following protein titration either with deuterated acetonitrile (ACN-d_3_) or 50 mM Na-phosphate buffer, pH 5.8, 5% D_2_O, in the range of 15–30% ACN-d_3_. The experimental time of each ^1^H–^15^N HSQC spectrum was 20 min; rCU remained soluble for all the titration experiments. The titration was carried out in two different ways: (I) to the protein initially solubilized in 30% ACN-d_3_/50 mM Na-phosphate buffer pH 5.8, 50 mM Na-phosphate buffer pH 5.8 was added to reduce the ACN-d_3_ percentage from 30% to 15%; (II) starting from the lyophilized protein in 50 mM Na-phosphate buffer containing 5% D_2_O, increasing aliquots of ACN-d_3_ were added to reach a final 30% ACN-d_3_ concentration. The two sets of spectra were consistent: they were superimposable at the same ACN:buffer proportion and CS variations followed a unique trend. Therefore, they were combined to obtain the complete titration from 0% to 30% ACN-d_3_. The CSPs were calculated using the following formula:

$$\Delta \delta_{NH}=\sqrt{\frac{(\Delta \delta_{H}{}^{2}+{\left(\Delta \delta_{N}/5\right)}^{2}\;}{2}}$$

where $\Delta \delta_{H}$ and $\Delta \delta_{N}$ are the differences in the CS observed between 0 and 30% ACN-d_3_ in the hydrogen and nitrogen dimensions, respectively.

The rationale of this experiment is to identify residues more sensitive to changes in the medium polarity.

### 4.5. Circular Dichroism Spectroscopy

Far-UV CD spectra (190–260 nm) have been recorded on a Jasco J-810 spectropolarimeter equipped with a Peltier system (PTC-348 WVI) for temperature control. Spectra have been collected at 25 °C with parameters as follows: 1 mm optical path length (light quartz cell), bandwidth 1 nm, speed 50 nm/min, response 8 s, and data pitch 0.5 nm. To improve the signal-to-noise ratio, the spectra reported are the average of four runs, each corrected by subtracting the respective blank. Ellipticity is reported as the mean residue molar ellipticity ([θ] 10^3^ degrees cm^2^ dmol^−1^).

CD spectra were acquired on four different samples:

→ 10 µM rCU dissolved in 30% ACN/70% H_2_O.
→ 10 µM rCU dissolved in 30% ACN/70% 50 mM Na-phosphate buffer pH 5.8.

rCU adsorbed on 150 nm diameter colloidal Teflon [58], kindly donated by Dr. M. Rossetti (Du Pont de Nemours, Deutschland). Before use, colloidal Teflon was filtered through glass wool to remove non-suspended material, and the Teflon concentration was determined by weighing the solution before and after solvent evaporation (0.19 g/mL). CD measurements were carried out at 9% surface coverage (2.85 μg CU/mg Teflon) to ensure all the protein molecules were adsorbed [59]. Each sample was prepared using 500 μL of Teflon in 50 mM Na-phosphate buffer, pH 5.8 and rCU, in 30% can/70% Na-phosphate buffer pH 5.8, to a final concentration of 10 µM. CD spectra of the adsorbed protein were carried out after overnight incubation at room temperature.

A solution of 10 µM rCU, 30% ACN, and 70% 50 mM Na-phosphate buffer pH 5.8 was dried on the external faces of the quartz cuvette. The suspension of CU was gently laid on one face of the cuvette, paying attention to covering the entire surface; subsequently, it was dried at room temperature under nitrogen flow for 3 h. The cuvette was then rotated, and the procedure was repeated on the opposite face.

## Data Availability

The data presented in this study are available in the article and supplementary material.

## Supplementary Materials

The following supporting information can be downloaded at https://www.mdpi.com/article/10.3390/ijms24032251/s1.

## References

[1] Wösten H.A., de Vocht M.L. (2000). Hydrophobins, the fungal coat unravelled. Biochim. Biophys. Acta.

[2] Linder M.B., Szilvay G.R., Nakari-Setälä T., Penttilä M.E. (2005). Hydrophobins: The protein-amphiphiles of filamentous fungi. FEMS Microbiol. Rev..

[3] Ball S.R., Kwan A.H., Sunde M. (2020). Hydrophobin Rodlets on the Fungal Cell Wall. Curr. Top. Microbiol. Immunol..

[4] Cai F., Zhao Z., Gao R., Chen P., Ding M., Jiang S., Fu Z., Xu P., Chenthamara K., Shen Q. (2021). The pleiotropic functions of intracellular hydrophobins in aerial hyphae and fungal spores. PLoS Genet..

[5] Wösten H.A.B., Scholtmeijer K. (2015). Applications of hydrophobins: Current state and perspectives. Appl. Microbiol. Biotechnol..

[6] Hakanpää J., Paananen A., Askolin S., Nakari-Setälä T., Parkkinen T., Penttilä M., Linder M.B., Rouvinen J. (2004). Atomic resolution structure of the HFBII hydrophobin, a self-assembling amphiphile. J. Biol. Chem..

[7] Dubey M.K., Jensen D.F., Karlsson M. (2014). Hydrophobins are required for conidial hydrophobicity and plant root colonization in the fungal biocontrol agent Clonostachys rosea. BMC Microbiol..

[8] Guzmán-Guzmán P., Alemán-Duarte M.I., Delaye L., Herrera-Estrella A., Olmedo-Monfil V. (2017). Identification of effector-like proteins in Trichoderma spp. and role of a hydrophobin in the plant-fungus interaction and mycoparasitism. BMC Genet..

[9] Quarantin A., Hadeler B., Kröger C., Schäfer W., Favaron F., Sella L., Martínez-Rocha A.L. (2019). Different Hydrophobins of Fusarium graminearum Are Involved in Hyphal Growth, Attachment, Water-Air Interface Penetration and Plant Infection. Front. Microbiol..

[10] Luti S., Bemporad F., Vivoli Vega M., Leri M., Musiani F., Baccelli I., Pazzagli L. (2020). Partitioning the structural features that underlie expansin-like and elicitor activities of cerato-platanin. Int. J. Biol. Macromol..

[11] Wösten H.A.B. (2001). Hydrophobins: Multipurpose Proteins. Annu. Rev. Microbiol..

[12] Bowden C.G., Hintz W.E., Jeng R., Hubbes M., Horgen P.A. (1994). Isolation and characterization of the cerato-ulmin toxin gene of the Dutch elm disease pathogen, Ophiostoma ulmi. Curr. Genet..

[13] Scala A., Comparini C., Tegli S., Mittempergher L., Scala F., del Sorbo G. (1994). Influence of fungal inoculum on cerato-ulmin production. Purification of cerato-ulmin and detection in elm sucker cuttings. Petria.

[14] Takai S. (1974). Pathogenicity and cerato-ulmin production in Ceratocystis ulmi. Nature.

[15] Kyte J., Doolittle R.F. (1982). A simple method for displaying the hydropathic character of a protein. J. Mol. Biol..

[16] Sbrana F., Fanelli D., Vassalli M., Carresi L., Scala A., Pazzagli L., Cappugi G., Tiribilli B. (2010). Progressive pearl necklace collapse mechanism for cerato-ulmin aggregation film. Eur. Biophys. J..

[17] Gómez-Pérez D., Chaudhry V., Kemen A., Kemen E. (2021). Amyloid Proteins in Plant-Associated Microbial Communities. Microb. Physiol..

[18] Askolin S., Linder M., Scholtmeijer K., Tenkanen M., Penttilä M., de Vocht M.L., Wösten H.A.B. (2021). Interaction and comparison of a class I hydrophobin from Schizophyllum commune and class II hydrophobins from *Trichoderma Reesei*. Biomacromolecules.

[19] Scala F., Bertelli E., Coppola L., Del Sorbo G., Tegli S., Scala A. (1997). Comparative determination of cerato-ulmin on cell surface and in mycelial extracts of pathogenic and non-pathogenic Ophiostoma species. Mycol. Res..

[20] Sherif S., Jones A.M.P., Shukla M.R., Saxena P.K. (2014). Establishment of invasive and non-invasive reporter systems to investigate American elm–Ophiostoma novo-ulmi interactions. Fungal Genet. Biol..

[21] Ren Q., Kwan A.H., Sunde M. (2014). Solution structure and interface-driven self-assembly of NC2, a new member of the Class II hydrophobin proteins. Proteins Struct. Funct. Bioinform..

[22] Ahuja I., Kissen R., Bones A.M. (2012). Phytoalexins in defense against pathogens. Trends Plant Sci..

[23] Biasini M., Bienert S., Waterhouse A., Arnold K., Studer G., Schmidt T., Kiefer F., Cassarino T.G., Bertoni M., Bordoli L. (2014). SWISS-MODEL: Modelling protein tertiary and quaternary structure using evolutionary information. Nucleic Acids Res..

[24] Laskowski R., Rullmann J.A., MacArthur M., Kaptein R., Thornton J. (1996). AQUA and PROCHECK-NMR: Programs for checking the quality of protein structures solved by NMR. J. Biomol. NMR.

[25] Ortega A., Amorós D., García de la Torre J. (2011). Prediction of Hydrodynamic and Other Solution Properties of Rigid Proteins from Atomic- and Residue-Level Models. Biophys. J..

[26] Shen Y., Delaglio F., Cornilescu G., Bax A. (2009). TALOS+: A hybrid method for predicting protein backbone torsion angles from NMR chemical shifts. J. Biomol. NMR.

[27] Bax A. (2003). Weak alignment offers new NMR opportunities to study protein structure and dynamics. Protein Sci..

[28] Niu M., Li Y., Wang C., Han K. (2018). RFAmyloid: A Web Server for Predicting Amyloid Proteins. Int. J. Mol. Sci..

[29] Conchillo-Solé O., de Groot N.S., Avilés F.X., Vendrell J., Daura X., Ventura S. (2007). AGGRESCAN: A server for the prediction and evaluation of “hot spots” of aggregation in polypeptides. BMC Bioinform..

[30] Lipari G., Szabo A. (1982). Model-free approach to the interpretation of nuclear magnetic resonance relaxation in macromolecules. 1. Theory and range of validity. J. Am. Chem. Soc..

[31] Fleming P.J., Fleming K.G. (2018). HullRad: Fast Calculations of Folded and Disordered Protein and Nucleic Acid Hydrodynamic Properties. Biophys. J..

[32] Landau M., Mayrose I., Rosenberg Y., Glaser F., Martz E., Pupko T., Ben-Tal N. (2005). ConSurf 2005: The projection of evolutionary conservation scores of residues on protein structures. Nucleic Acids Res..

[33] Temple B., Horgen P.A. (2000). Biological roles for cerato-ulmin, a hydrophobin secreted by the elm pathogens, *Ophiostoma ulmi* and *O. novo-ulmi*. Mycologia.

[34] Dodds P.N., Rathjen J.P. (2010). Plant immunity: Towards an integrated view of plant–pathogen interactions. Nat. Rev. Genet..

[35] Newman M.-A., Sundelin T., Nielsen J.T., Erbs G. (2013). MAMP (microbe-associated molecular pattern) triggered immunity in plants. Front. Plant Sci..

[36] Lombardi L., Faoro F., Luti S., Baccelli I., Martellini F., Bernardi R., Picciarelli P., Scala A., Pazzagli L. (2013). Differential timing of defense-related responses induced by cerato-platanin and cerato-populin, two non-catalytic fungal elicitors. Physiol. Plant..

[37] Pazzagli L., Seidl-Seiboth V., Barsottini M., Vargas W.A., Scala A., Mukherjee P.K. (2014). Cerato-platanins: Elicitors and effectors. Plant Science.

[38] Del Sorbo G., Scala A., Scala F., Tegli S. (2002). Cerato-Ulmin, a Toxin Produced by the Pathogens of the Dutch Elm Disease. Advances in Microbial Toxin Research and Its Biotechnological Exploitation.

[39] Ruocco M., Lanzuise S., Lombardi N., Woo S.L., Vinale F., Marra R., Varlese R., Manganiello G., Pascale A., Scala V. (2015). Multiple Roles and Effects of a Novel Trichoderma Hydrophobin. Mol. Plant-Microbe Interact..

[40] Fraczkiewicz R., Braun W. (1998). Exact and efficient analytical calculation of the accessible surface areas and their gradients for macromolecules. J. Comput. Chem..

[41] Zhang X., Kirby S.M., Chen Y., Anna S.L., Walker L.M., Hung F.R., Russo P.S. (2018). Formation and elasticity of membranes of the class II hydrophobin Cerato-ulmin at oil-water interfaces. Colloids Surf. B Biointerfaces.

[42] Carresi L., Pantera B., Zoppi C., Cappugi G., Oliveira A.L., Pertinhez T.A., Spisni A., Scala A., Pazzagli L. (2006). Cerato-platanin, a phytotoxic protein from Ceratocystis fimbriata: Expression in Pichia pastoris, purification and characterization. Protein Expr. Purif..

[43] Baccelli I., Lombardi L., Luti S., Bernardi R., Picciarelli P., Scala A., Pazzagli L. (2014). Cerato-Platanin Induces Resistance in Arabidopsis Leaves through Stomatal Perception, Overexpression of Salicylic Acid- and Ethylene-Signalling Genes and Camalexin Biosynthesis. PLoS ONE.

[44] Delaglio F., Grzesiek S., Vuister G., Zhu G., Pfeifer J., Bax A. (1995). NMRPipe: A multidimensional spectral processing system based on UNIX pipes. J. Biomol. NMR.

[45] Johnson B.A. (2004). Using NMRView to Visualize and Analyze the NMR Spectra of Macromolecules. Methods Mol. Biol..

[46] Jones J., Wilkins D., Smith L., Dobson C. (1997). Characterisation of protein unfolding by NMR diffusion measurements. J. Biomol. NMR.

[47] Wilkins D.K., Grimshaw S.B., Receveur V., Dobson C.M., Jones J.A., Smith L.J. (1999). Hydrodynamic Radii of Native and Denatured Proteins Measured by Pulse Field Gradient NMR Techniques. Biochemistry.

[48] Aran M., Smal C., Pellizza L., Gallo M., Otero L.H., Klinke S., Goldbaum F.A., Ithurralde E.R., Bercovich A., Mac Cormack W.P. (2014). Solution and crystal structure of BA42, a protein from the Antarctic bacterium B izionia argentinensis comprised of a stand-alone TPM domain. Proteins Struct. Funct. Bioinform..

[49] Baroni F., Gallo M., Pazzagli L., Luti S., Baccelli I., Spisni A., Pertinhez T.A. (2021). A mechanistic model may explain the dissimilar biological efficiency of the fungal elicitors cerato-platanin and cerato-populin. Biochim. Biophys. Acta (BBA)-Gen. Subj..

[50] Ottiger M., Delaglio F., Bax A. (1998). Measurement ofJand Dipolar Couplings from Simplified Two-Dimensional NMR Spectra. J. Magn. Reson..

[51] Hansen M.R., Mueller L., Pardi A. (1998). Tunable alignment of macromolecules by filamentous phage yields dipolar coupling interactions. Nat. Struct. Biol..

[52] Zweckstetter M., Bax A. (2000). Prediction of Sterically Induced Alignment in a Dilute Liquid Crystalline Phase: Aid to Protein Structure Determination by NMR. J. Am. Chem. Soc..

[53] Cornilescu G., Marquardt J.L., Ottiger M., Bax A. (1998). Validation of Protein Structure from Anisotropic Carbonyl Chemical Shifts in a Dilute Liquid Crystalline Phase. J. Am. Chem. Soc..

[54] Hwang T.L., Van Zjil P.C.M., Mori S. (1998). Accurate quantification of water amide proton exchanges rates using the Phase-Modulated CLEAN chemical EXchange (CLEANEX-PM) approach with a Fast-HSQC (FHSQC) detection scheme. J. Biomol. NMR.

[55] Kay L.E., Torchia D.A., Bax A. (1989). Backbone dynamics of proteins as studied by 15N inverse detected heteronuclear NMR spectroscopy: Application to staphylococcal nuclease. Biochemistry.

[56] Noguera M.E., Aran M., Smal C., Vazquez D.S., Herrera M.G., Roman E.A., Alaimo N., Gallo M., Santos J. (2017). Insights on the conformational dynamics of human frataxin through modifications of loop-1. Arch. Biochem. Biophys..

[57] Dosset P., Hus J.C., Blackledge M., Marion D. (2000). Efficient analysis of macromolecular rotational diffusion from heteronuclear relaxation data. J. Biomol. NMR.

[58] Lumsdon S.O., Green J., Stieglitz B. (2005). Adsorption of hydrophobin proteins at hydrophobic and hydrophilic interfaces. Colloids Surf. B Biointerfaces.

[59] de Vocht M.L., Scholtmeijer K., van der Vegte E.W., de Vries O.M.H., Sonveaux N., Wösten H.A.B., Ruysschaert J.-M., Hadziioannou G., Wessels J.G.H., Robillard G.T. (1998). Structural Characterization of the Hydrophobin SC3, as a Monomer and after Self-Assembly at Hydrophobic/Hydrophilic Interfaces. Biophys. J..

