# Supplementary material for: Plant Defense Elicitation by the Hydrophobin Cerato-Ulmin and Correlation with Its Structural Features
Source: Int J Mol Sci. 2023 Jan 23;24(3):2251. doi: 10.3390/ijms24032251 (PMC9916430; doi:10.3390/ijms24032251)

Plant Defense Elicitation by the Hydrophobin Cerato-Ulmin and Correlation with Its Structural Features

**Mariana Gallo [1,†], Simone Luti [2,†], Fabio Baroni [1,‡], Ivan Baccelli [3], Eduardo Maffud Cilli [4], Costanza Cicchi [2],
Manuela Leri [2], Alberto Spisni [1,*], Thelma A. Pertinhez [1,§] and Luigia Pazzagli [2,§]**

TABLE AND FIGURES OF SUPPLEMTARY MATERIAL

Table S1. Experimental RDCs (D_{HN}^{obs} column) versus calculated RDCs (D_{HN}^{calc} column). The third column reports D_{HN}^{obs} - D_{HN}^{calc} for each residue considered in the analysis. According to equation (1) the Q factor is estimated to be 0.551.

	D_{HN}^{obs}	D_{HN}^{calc}	ΔD_{HN}			D_{HN}^{obs}	D_{HN}^{calc}	ΔD_{HN}
**SER5**	0.7	0.4673	0.2327		**SER45**	1	0.6743	0.3257
**ASP6**	1.9	1.612	0.288		**GLN46**	0	-0.03775	0.03775
**SER7**	1	0.4702	0.5298		**PHE47**	0	-0.5417	0.5417
**TYR8**	0.5	0.9436	-0.4436		**GLN48**	1	0.4778	0.5222
**ASP9**	0.5	-0.277	0.777		**ALA49**	1	0.711	0.289
**CYS11**	1	0.937	0.063		**SER50**	0.5	0.2077	0.2923
**THR12**	1	0.351	0.649		**CYS51**	0.5	-0.1234	0.6234
**GLY13**	0	-0.5811	0.5811		**VAL52**	0.5	0.1268	0.3732
**LEU14**	1.6	0.9994	0.6006		**ALA53**	1.1	0.8006	0.2994
**GLN16**	0.6	0.7041	-0.1041		**ASP54**	0	0.0349	-0.0349
**LYS17**	0	0.6011	-0.6011		**GLY55**	0	0.0432	-0.0432
**SER18**	1.6	1.3131	0.2869		**GLY56**	1	0.7525	0.2475
**GLN20**	0	0.7793	-0.7793		**ARG57**	0.5	-0.1629	0.6629
**CYS21**	0.5	-0.2256	0.7256		**SER58**	1.1	0.2855	0.8145
**CYS22**	0.5	-0.0279	0.5279		**ALA59**	0.6	-0.1592	0.7592
**ASN23**	0.5	-0.1248	0.6248		**ARG60**	0.5	0.3498	0.1502
**THR24**	0.5	0.7175	-0.2175		**CYS62**	0	0.7537	-0.7537
**ASP25**	1.6	1.7177	-0.1177		**THR63**	0	0.579	-0.579
**ILE26**	0.5	0.1553	0.3447		**LEU64**	1	1.0377	-0.0377
**LEU27**	1.1	0.3804	0.7196		**SER65**	0.6	1.1189	-0.5189
**GLY28**	0	-0.6621	0.6621		**LEU66**	0.5	0.0774	0.4226
**ALA30**	0.5	0.4035	0.0965		**LEU67**	0	0.3076	-0.3076
**ASN31**	1	1.1342	-0.1342		**GLY68**	0.3	0.1413	0.1587
**LEU32**	2	1.1632	0.8368		**LEU69**	0.5	1.0275	-0.5275
**ASP33**	1.5	1.4575	0.0425		**ALA70**	0	0.2758	-0.2758
**CYS34**	1.6	1.4112	0.1888		**LEU71**	0.5	0.3528	0.1472

**HIS35**	1.6	0.7185	0.8815		**VAL72**	1.5	1.1362	0.3638
**GLY36**	0.5	-0.0364	0.5364		**CYS73**	0	0.341	-0.341
**SER39**	0.5	0.1124	0.3876		**THR74**	0	0.244	-0.244
**VAL40**	1	0.5153	0.4847		**ASP75**	0.5	0.8241	-0.3241
**THR42**	0.6	0.5301	0.0699		**GLY78**	0.6	0.332	0.268
**SER43**	0.5	0.0762	0.4238		**ILE79**	0.5	0.0899	0.4101

Figure S1. ^{1}H-^{15}N-HSQC spectrum of U-^{15}N-rCU in 30% ACN-d_3:70% 50 mM phosphate buffer pH 5.8. at 15 °C. Negative numbers refer to the residues of the non-native additional EAEA N-terminal sequence

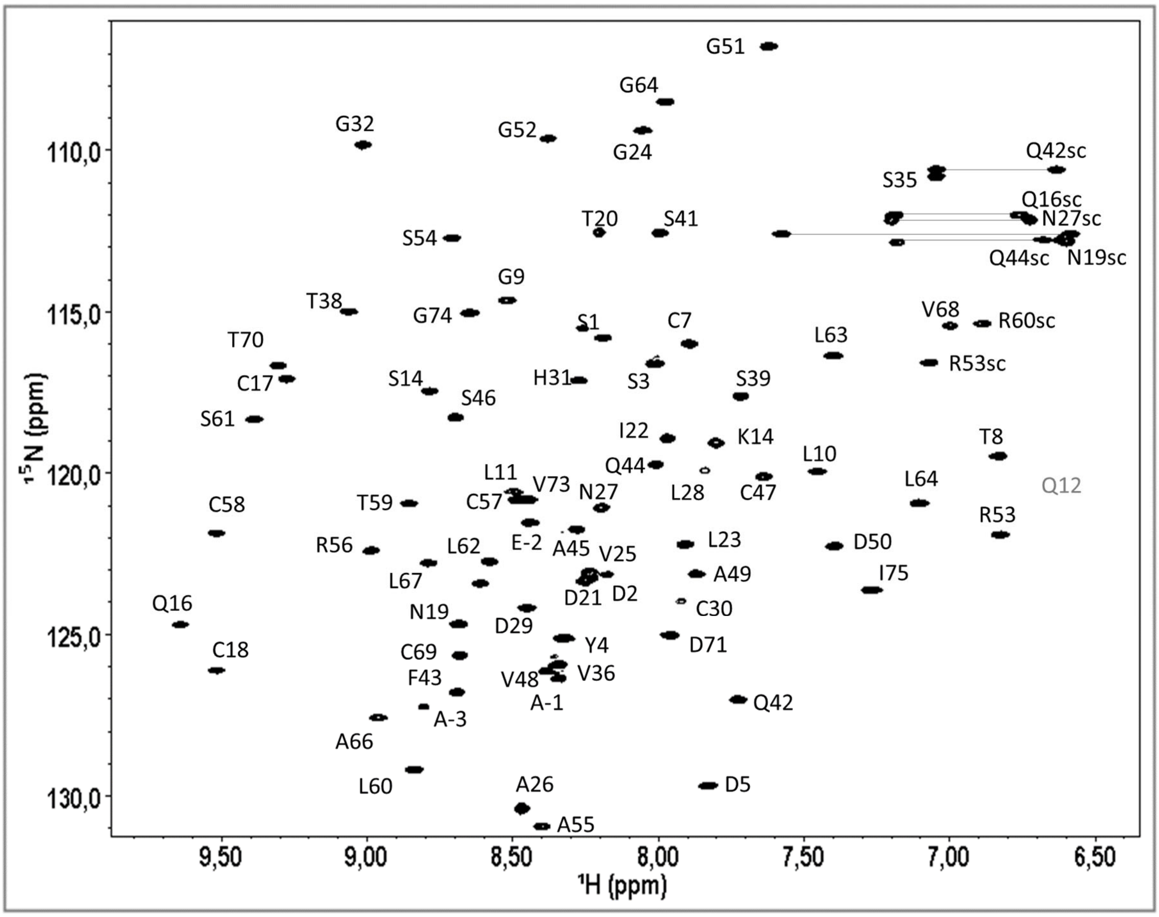

Figure S2. Solvent accessibility data: H/D exchange and CLEANEX-PM.

Amide protons with low H/D exchange rates. that remain visible after more than 4000 min in the ^{1}H-^{15}N HSQC spectrum upon D_2O resuspension (black bars) and CLEANEX-PM data. reported as the ratio of the CLEANEX-PM/HSQC peak intensities (grey bars).

A cartoon of the topology of the CU model is reported in the upper part of the figure.

The secondary structure elements are coincident with regions protected from solvent exchange. with lower H/D exchange rates and amide low water-NOE correlations. Prolines (missing signals) are marked with an asterisk (*).

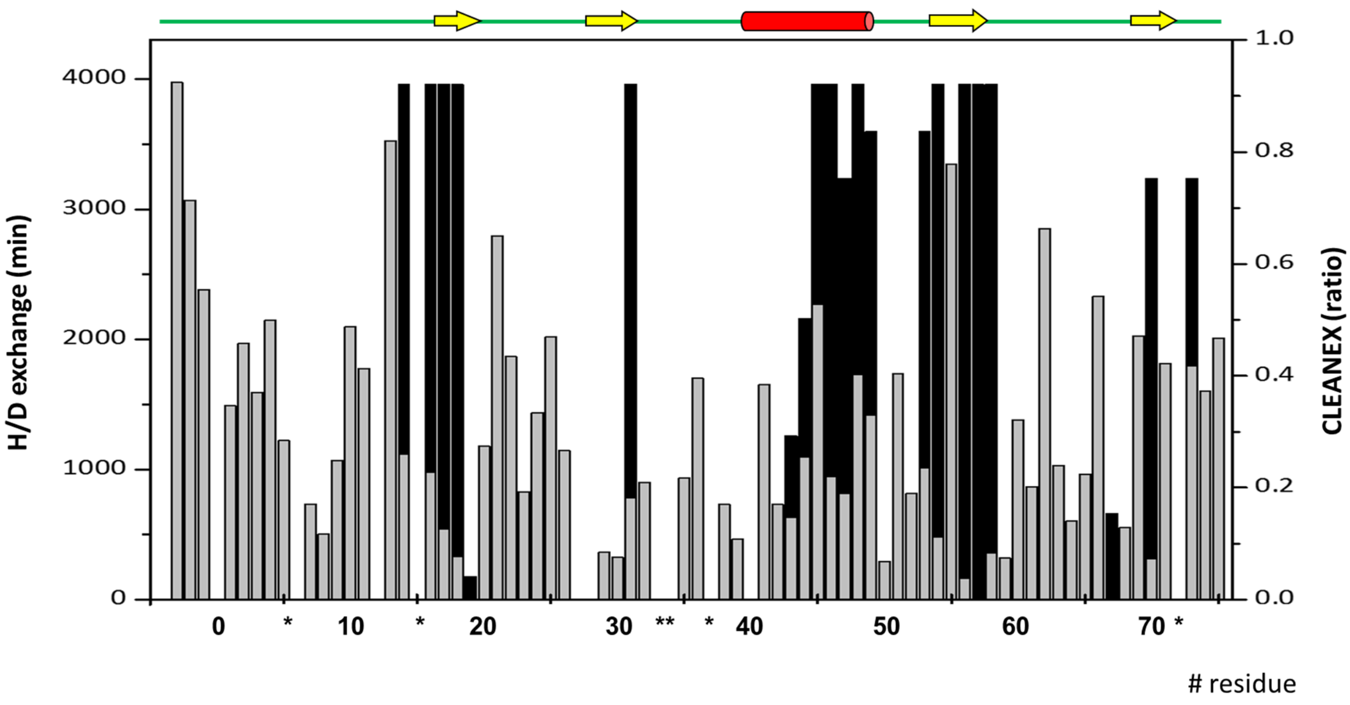

Figure S3. Experimental RDCs (D_{HN}^{obs}) versus calculated RDCs (D_{HN}^{calc}).

Experimental validation of CU's model. The correlation plot shows the degree of agreement between experimentally measured RDCs and those calculated from the calculated homology model. Q factor is estimated to be 0.56.

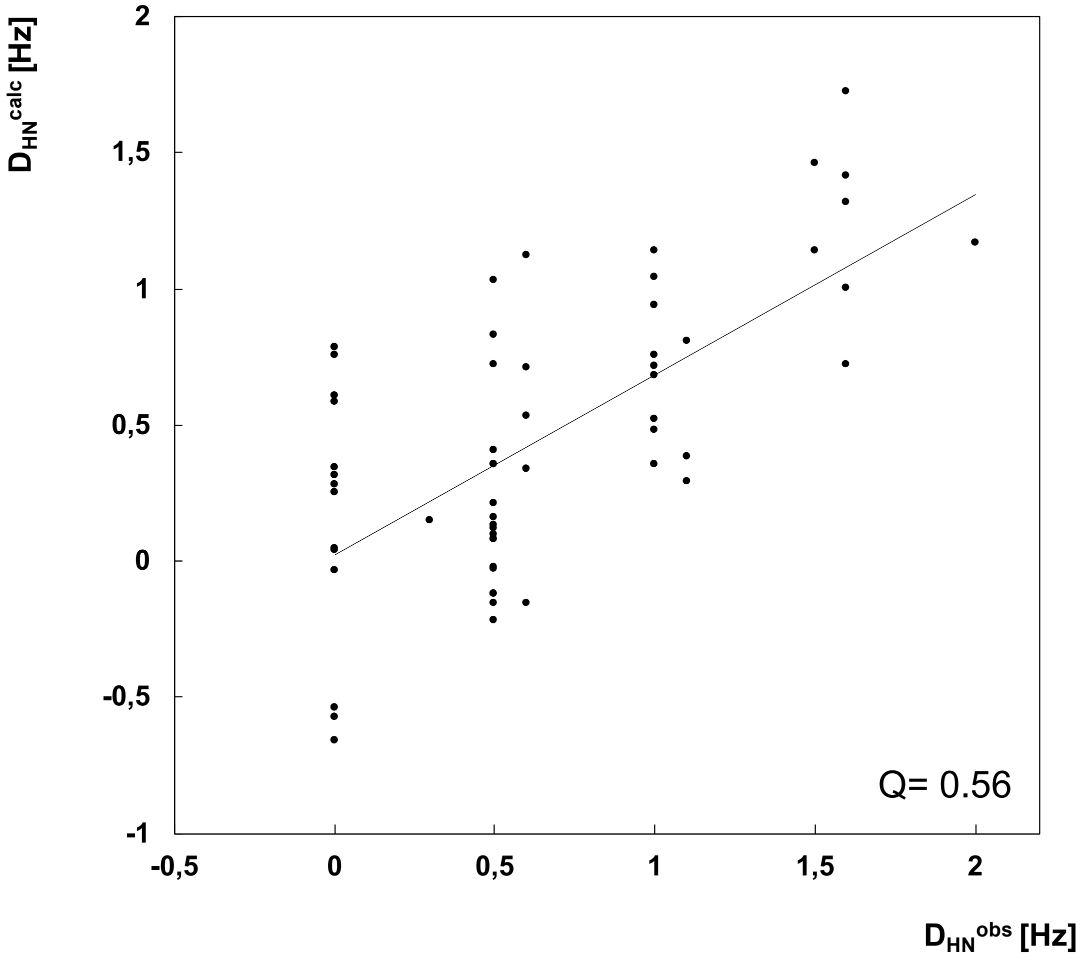

Figure S4. CU residues with aggregation tendency. The CU aggregation profile was analyzed using the AGGRESCAN server. The probability of aggregation propensity is plotted against the protein sequence (A) and mapped onto the 3D CU model (B).
A cartoon of the topology of the CU model is reported in the upper part of the figure.

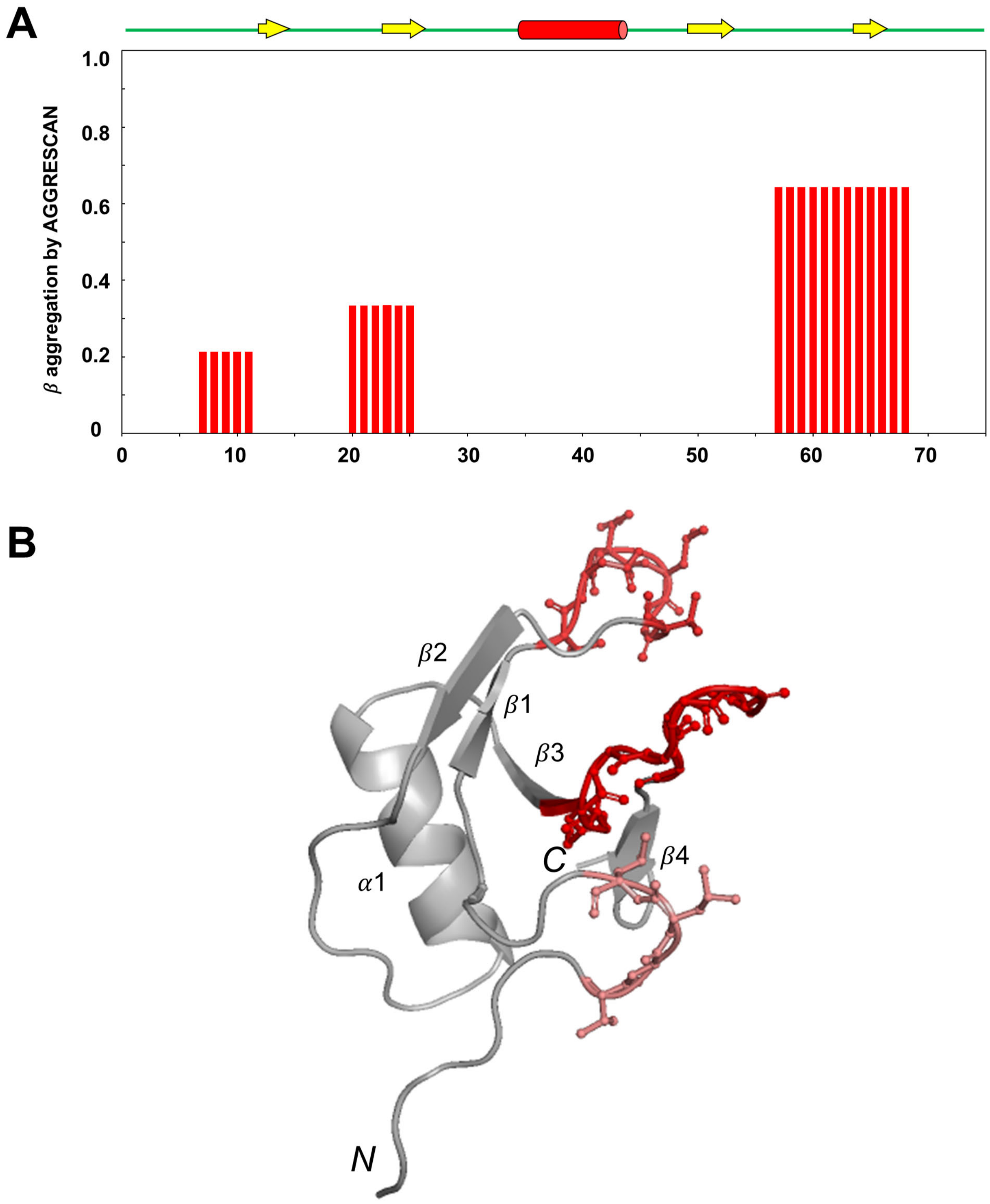

Figure S5. ^{15}N-NMR dynamics experiments.

R_1. R_2. and hetNOE of rCU 700 µM in 30% ACN-d_3:70% 50 mM phosphate buffer pH 5.8. at 15 °C. red lines indicate the average values of the relaxation parameters.

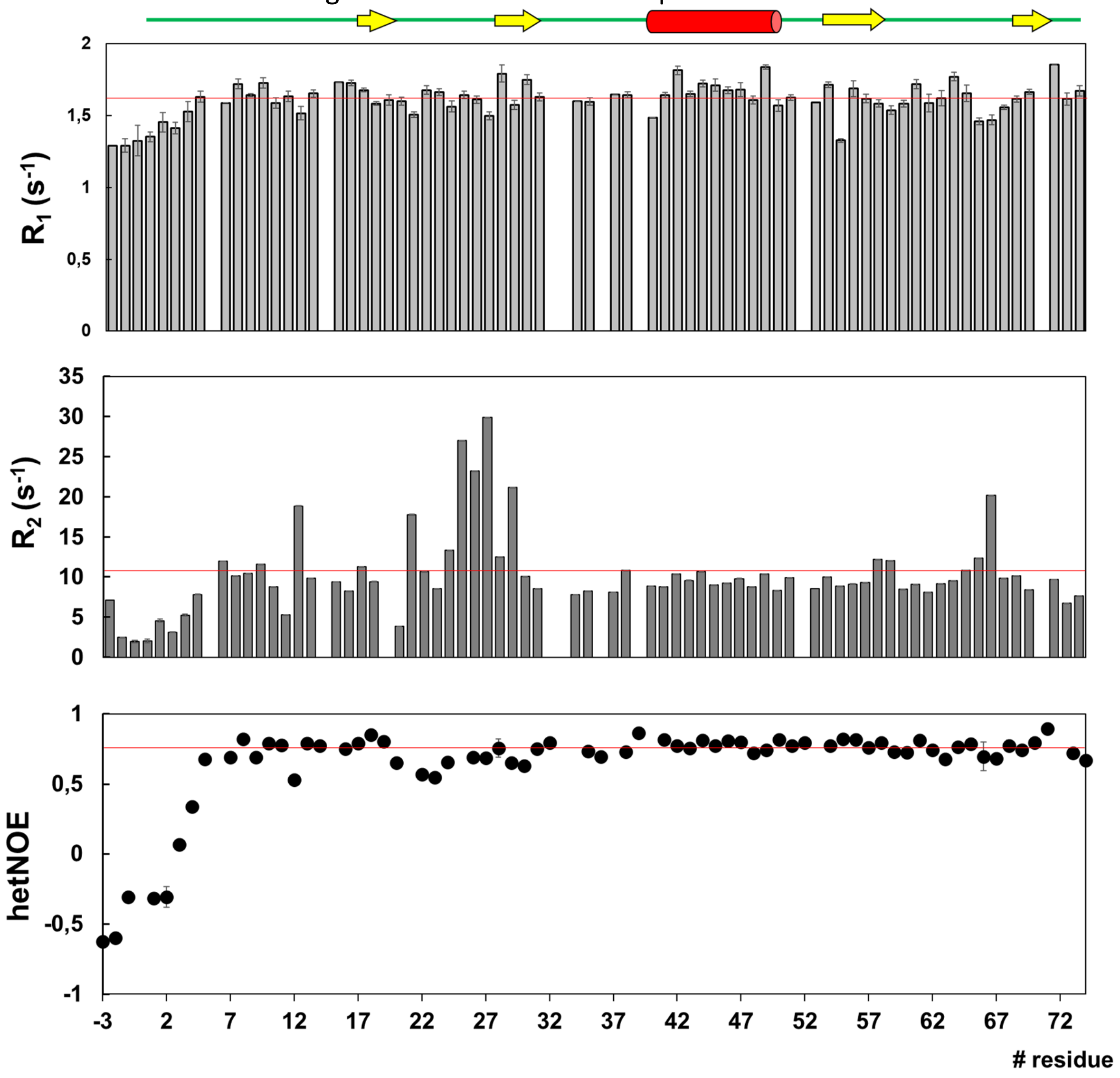

Figure S6. Conserved residues and hydrophobic patches.
Conserved residues amongst class-II hydrophobins calculated with the ConSurf server. Conserved residues are coloured on the CU sequence (A) and on the 3D model (B) (dark blue for highly conserved amino acids and progressively lighter colours for medium and low conserved).
C) Surface electrostatic potential in the CU model calculated with Pymol. showing the hydrophobic character of the β1β2- and β3β4-loops.

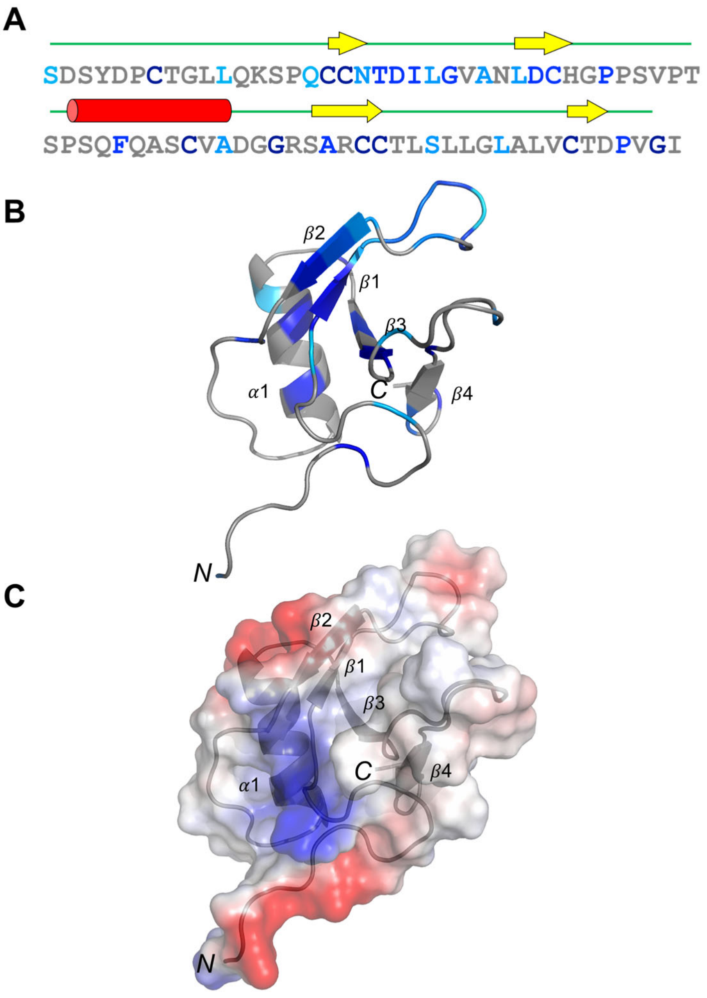

Figure S7. Chemical shift titration experiments.
Section of the ^{1}H-^{15}N HSQC spectrum of rCU in phosphate buffer. showing chemical shift perturbations upon addition of increasing amounts of ACN-d_3.

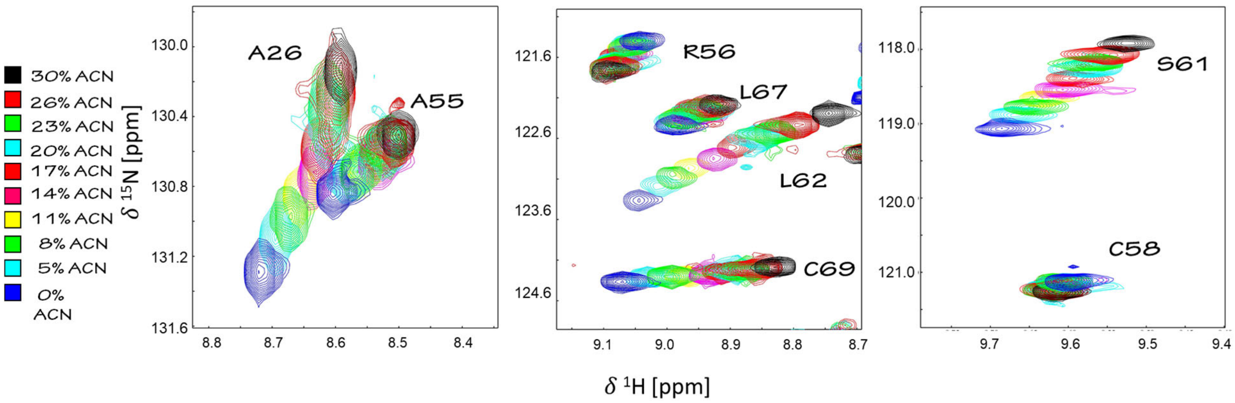

Supplement: Supplementary file 1 [file ijms-24-02251-s001.zip › ijms-2174381-supplementary.pdf]
